# Shake-table testing of unreinforced fly ash brick masonry model with unreinforced elastomeric isolator

**DOI:** 10.1038/s41598-025-04549-5

**Published:** 2025-06-04

**Authors:** Zoheb Nawaz Md, Mohan S. C., Sri Kalyana Rama Jyosyula

**Affiliations:** 1 Department of Civil Engineering, BITS Pilani Hyderabad Campus, Hyderabad, Telangana 500078 India; 2 Department of Civil Engineering, Ecole Centrale School of Engineering, Mahindra University, Hyderabad, Telangana 500043 India

**Keywords:** Unreinforced elastomeric isolator, Shake table-testing, Unreinforced fly ash brick masonry, Base isolation, Low-cost isolation system, Civil engineering, Natural hazards

## Abstract

Seismic isolation techniques are widely used to mitigate earthquake-induced damage in structures; however, their application in low-cost unreinforced masonry (URM) buildings remains limited. This study investigates the effectiveness of Unreinforced Elastomeric Isolators (UEIs) for improving the seismic resilience of URM structures through comprehensive shake-table testing. A half-scale, single-story masonry model was constructed based on similitude laws and tested under both isolated and non-isolated conditions. The UEI was designed and experimentally characterized before implementation. Free and forced vibration tests were conducted to determine the natural frequencies of the model, followed by shake table tests simulating seven earthquake ground motions. Results indicate that the non-isolated model experienced significant acceleration amplification, whereas the isolated model demonstrated reduced roof acceleration and overall seismic force transmission. Despite observable rocking motion in the isolated case, the total roof displacement was lower than in the non-isolated scenario. Comparisons of base shear and overturning moments further confirmed the improved seismic performance of the UEI-supported model. These findings highlight UEIs as a promising, cost-effective solution for seismic protection of masonry structures, paving the way for further optimization and real-world implementation.

**Supplementary Information:**

The online version contains supplementary material available at 10.1038/s41598-025-04549-5.

## Introduction

Despite advancements in the construction industry, non-engineered constructions (NEC) remain prevalent in many regions, including those prone to high seismic activity. These structures often rely on locally available materials such as brick, stone, and adobe^1^. Examples of NEC include masonry structures, confined masonry structures, and wooden buildings. Confined masonry buildings feature masonry walls supported by tie beams and tie columns made of reinforced concrete (RC), steel, or wood^2–4^. However, due to the absence of engineered design and reliance on traditional construction practices, these structures are highly vulnerable to collapse or severe damage during moderate to strong earthquakes^5–8^.

In developing countries, unreinforced masonry (URM) structures are widely constructed using clay bricks and cement mortar, with occasional use of mud mortar or concrete blocks. Reinforced concrete is commonly employed for roofs and floors. Despite their well-documented low seismic capacity^9^, URM buildings continue to represent a substantial portion of the building stock in these regions^7,8,10^. The collapse of URM structures during seismic events has resulted in significant casualties and economic losses^8,11^. Countries such as India, Pakistan, El Salvador, Mexico, Indonesia, Ecuador, the Philippines, and Nepal are particularly vulnerable. For instance, the 2006 Yogyakarta earthquake damaged approximately 59% of masonry buildings, while the 2005 Kashmir earthquake led to the failure of 99% of URM structures^12,13^. Inadequate design and the lack of seismic considerations have contributed to extensive damage or total collapse in buildings in India and Pakistan^13^. While completely replacing these structures with modern earthquake-resistant buildings is financially impractical, enhancing their seismic performance through improved structural detailing is essential for mitigating earthquake-induced damage^14,15^. In response, efforts are underway in rural areas of developing countries to reinforce URM structures by incorporating tie beams at various floor levels^16^.

Experimental studies have consistently shown that URM buildings are particularly vulnerable to seismic damage, with in-plane failures driven by shear at the lower storeys and out-of-plane failures, especially in gable walls, initiating at upper levels due to high acceleration demands^17,18^. Although retrofitting techniques such as horizontal ties and RC bands can enhance lateral resistance and prevent wall separation^19^, they are often invasive, costly. Given these observed vulnerabilities, strategies that reduce the seismic demand transmitted to the structure, such as base isolation, offer a compelling alternative.

Seismic isolation^20^ is an effective technique for reducing seismic forces by introducing a flexible layer between the foundation and superstructure of a building. This approach alters the building’s dynamic characteristics, lowering its fundamental frequency and reducing the energy transferred from the earthquake. Because of the significant expense associated with isolation systems, they are generally reserved for essential infrastructure, including hospitals, historical buildings, bridges, and emergency centers. Traditional seismic isolators generally use elastomeric or frictional devices. Recent developments include fiber-reinforced elastomeric isolators (FREIs)^21–27^, which are lightweight, easy to install, and often configured without internal steel shims. Efforts have been made to further reduce the cost of FREIs by developing eco-friendly versions using recycled rubber (RR-FREIs)^28– 30^.

Geotechnical seismic isolation is another low-cost method that can be employed with minimal tools and resources^31^. Researchers explored the use of a geotechnical isolation system that employs sliding over a pebble-stone layer and is designed specifically for low-rise structures^32^. Similarly, the impact of rubber-soil mixture layers on buildings has been evaluated^33^. Tsiavos et al.^34,35^ have presented and experimentally tested highly efficient, sliding-based seismic isolation systems at a large scale for low-cost seismic protection in developing countries based on materials locally available in these countries. The ease of manufacturing and accessibility of natural resources make these geotechnical isolation methods appealing to developing nations. Recent studies have investigated various seismic protection methods for construction in developing regions^36^.

Previous research has extensively examined the seismic behavior of URM structures in the context of various isolation systems. In a previous study, the authors devised an economic isolation method utilizing unreinforced rubber^37^. A detailed numerical investigation was carried out to evaluate the effectiveness of the UEI for the masonry buildings. Along with this, the authors have carried out a detailed experimental investigation on the UEI^38^. This approach presents a significant opportunity for developing countries and resource-limited communities seeking enhanced seismic protection for low-rise masonry buildings. To further validate the effectiveness of the unreinforced elastomeric isolator, the authors conducted shake-table testing on a half-scale masonry model. It is important to note that this study is primarily focused on new construction applications. Future work is planned to explore the potential of UEIs for retrofitting existing structures. The experimental methodology adopted in this study aligns with established practices reported in the literature^39,40^.

Initially, a half-scale masonry model was constructed in accordance with the actuator specifications and similitude laws. Subsequently, an isolator was designed based on this masonry model and evaluated for its vertical and horizontal stiffness. The masonry model was then mounted on the UEI and connected to the shake table. Both free vibration and forced vibration analyses were performed on the isolated model. Shake table tests were conducted for isolated and non-isolated models using seven different ground motions. The results from these tests were compared to assess their performance.

## Experimental setup

An experimental study was conducted on a half-scale model of a single-story URM building. Fly ash brick masonry, a commonly used construction material in the region of the test site, was selected for the test model. The shake table used in the experiment measures 2 m × 3 m and has a load capacity of 12 t. The dimensions of the test model were determined based on these specifications. The unreinforced elastomeric isolator (UEI) was designed to support the expected gravity load of the test model. The detailed design of the UEI is discussed in the following section (*see description of UEI*). Nawaz et al.^38^ thoroughly analyzed the performance of the UEI under cyclic loading. Detailed information about the test model and the UEI is provided in the following subsection.

### Test model description

A scale of 1:2 was chosen to balance the practical constraints of shake table size (2 m × 3 m) and payload capacity (12 t) while ensuring representative dynamic behavior. This scale allows for accurate replication of seismic responses while maintaining the manufacturability of UEIs with appropriate stiffness characteristics. The similitude laws, as outlined by Harris and Sabnis^41^, were applied to determine the scaled dimensions of the masonry model and the additional mass required for gravity load simulation. Table 1 presents the scale relationships derived for the relevant parameters in this study.

**Table 1:** Similitude relationship.

Parameters	Scale	Half scale Model
Length	S	2
Mass	S^3^	8
Displacement	S	2
Time	$\:\sqrt{s}$	1.414
Acceleration	S	2

**Fig. 1:**
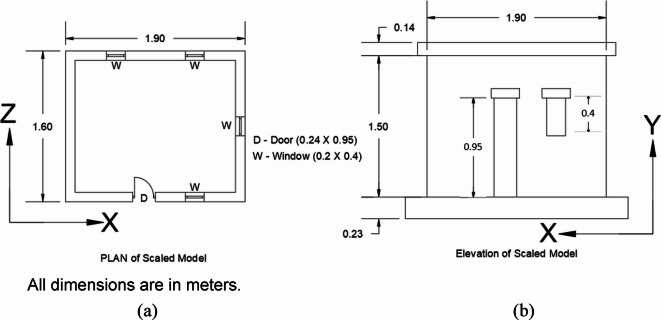
Geometrical details of the half-scaled masonry model (a) Plan, (b) Elevation.

Following the similitude laws, the scaled model was carefully designed. Figure 1 illustrates the plan and elevation of the model, while Table 2 provides the geometrical details. The weight of the full-scale prototype is 20.7 tons, while the total weight of the scaled model is 2.07 tons. However, according to similitude laws, the mass was expected to be approximately 2.6 t. To adhere to the mass similitude law, the slab thickness was increased from 50 mm to 140 mm, resulting in a 180% mass increase.

**Fig. 2:**
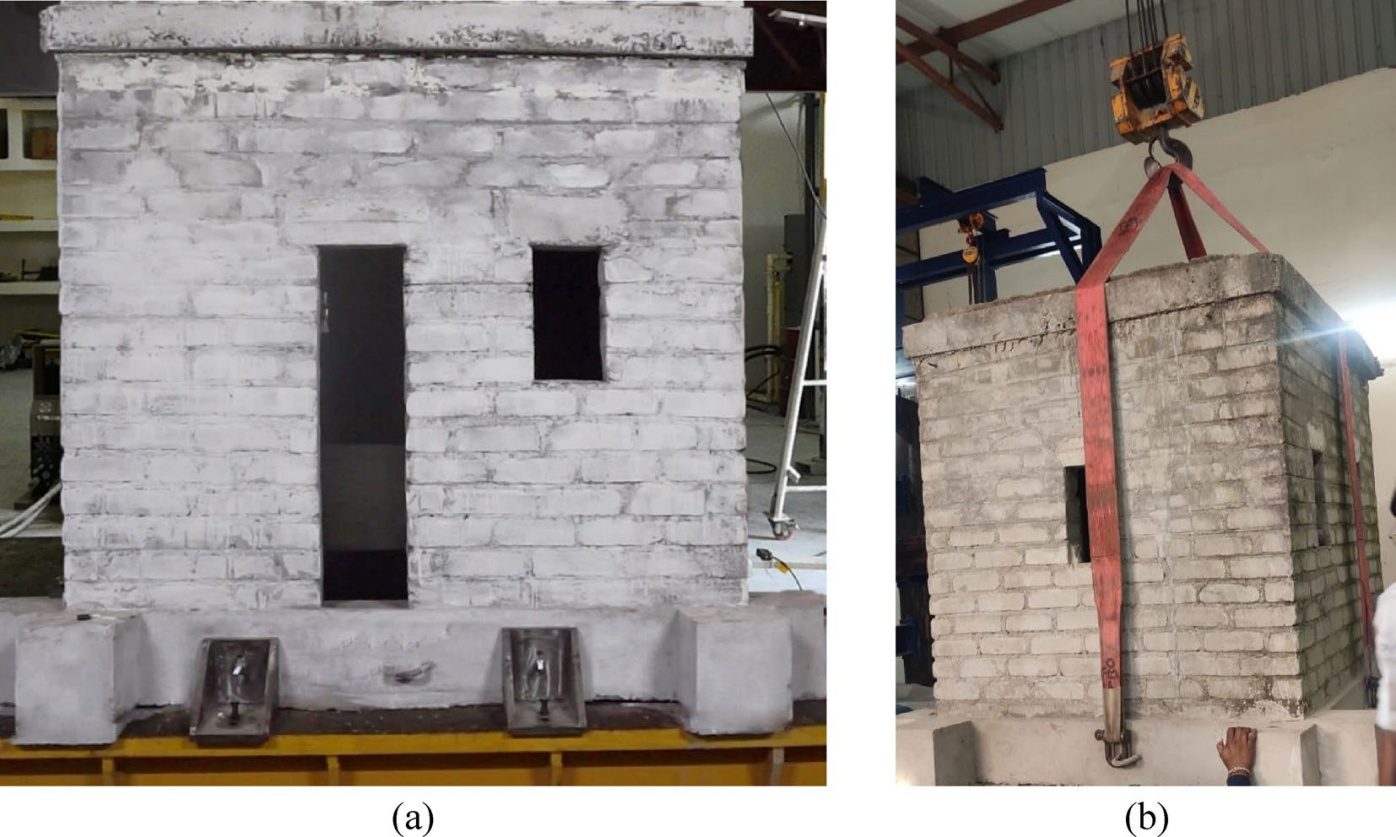
(a) constructed half-scaled masonry model; (b) Hoisting process.

The masonry model was constructed using fly ash bricks bonded with cement mortar in a 1:3 ratio. The walls were laid in a stretcher bond pattern with a mortar joint thickness of 12 mm. The model was constructed on a plinth (referred to as the top plinth throughout this paper) with dimensions of 230 mm × 230 mm, allowing it to be supported by the UEI. The mechanical properties of the masonry units and the masonry prism, determined through tests conforming to the relevant codes, are presented in Table 3. The model was cast outside the Advanced Structural Engineering Laboratory at BITS-Pilani, Hyderabad campus. To facilitate moving the model into the lab, hooks were embedded on all four sides of the plinth during casting, and the plinth beam was specifically designed to withstand the lifting process. Figure 2 shows the constructed half-scale masonry model and the hoisting process.

**Table 2:** Description of the half-scaled model.

Parameters	Scaled model
Length of the model (m)	1.9
Width of the model (m)	1.6
Thickness of the wall (m)	0.1
Height of the model (m)	1.5
Thickness of the slab (m)	0.14
Dimensions of the Plinth beam	0.23 × 0.23
Weight of the model (t)	2.07
Weight after increasing the thickness (t)	2.75

**Table 3:** Mechanical properties of the masonry components.

Characteristics	Value	Code reference
Fly ash brick compressive strength	26.60 MPa	ASTM C67-13
Mortar (1:3) compressive strength	6.96 MPa	ASTM C109/C109M-13
Five-stack masonry prism compressive strength	7.72 MPa	ASTM C1314-14
Modulus of elasticity of five-stack masonry prism	5244 MPa	ASTM C1314-14
Density of masonry	1580 kg/m^3^	ASTM C140
Modulus of elasticity of concrete	24,768 MPa	BIS (2000)
Density of concrete	2400 kg/m^3^	BIS (1987b)

### Description of UEI

In this study, the UEI is designed as the base isolation system for the half-scale masonry model. The estimated total weight of the half-scale model is 2.75 t. When including the plinth beam, the total weight increases to 3.8 t. To distribute this load effectively and ensure uniform support and isolation behavior, the model was mounted on four UEIs, positioned at each corner. Each isolator was designed to support approximately 0.95 t (9.5 kN). To ensure structural stability, it was verified that the vertical load on each UEI, i.e., approximately 9.5 kN, remained well below the critical buckling load of the isolator, thereby preventing potential instability under gravity loading^38^. The dimensions of the model isolator are calculated using Eqs. 1 and 2, as proposed by Naem and Kelly^17^ for achieving the isolation period of the test model as 0.95 s [(fundamental period of prototype isolated building = 1.35 s)/√Scale factor] at 100% shear strain for the desired efficacy in seismic isolation. This time period was selected to ensure the isolation frequency remains significantly lower than the predominant frequency range of the input ground motions, effectively filtering high-frequency seismic energy.

1 $$\:{K}_{h}=M.{\left(\frac{2\pi\:}{{T}_{h}}\right)}^{2}.$$

The target equivalent lateral stiffness is determined to be 47.834 N/mm using Eq. 1. Given a shear modulus ($\:G$) of 0.35 MPa, and the rubber thickness ($\:{t}_{r}$) of 40 mm, the plan area dimension ($\:A$) of the rubber is determined using Eq. 2.

2 $$\:A=\frac{{K}_{h}.{t}_{r}}{G}.$$

**Fig. 3:**
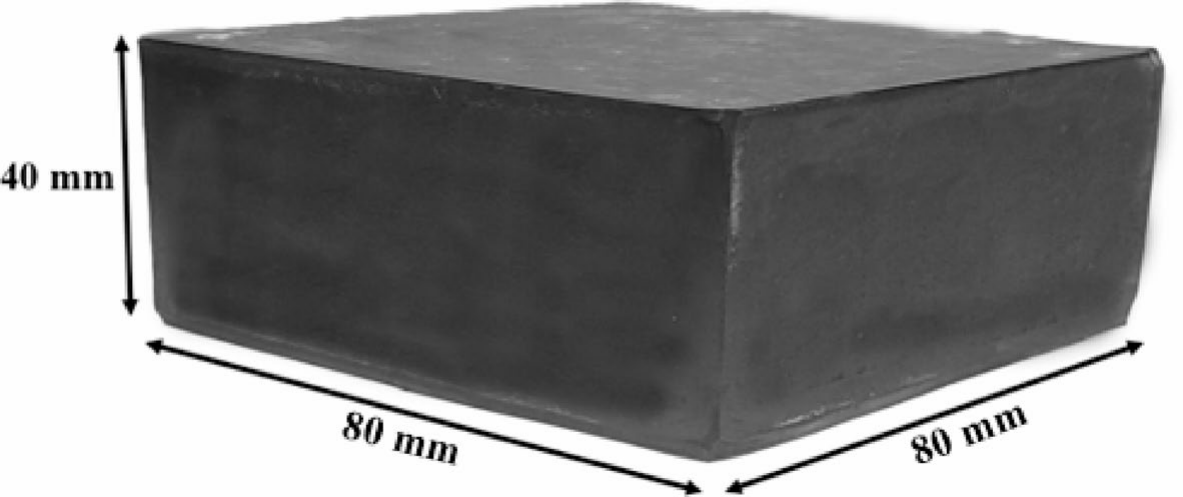
Manufactured UEI using natural rubber.

From the equation, the rubber area is determined as 77.46 mm × 77.46 mm. For ease of manufacturing, these dimensions are rounded to 80 mm × 80 mm. Figure 3 depicts the manufactured UEI.

## Experimental testing of UEI

### Dynamic compression test

**Fig. 4:**
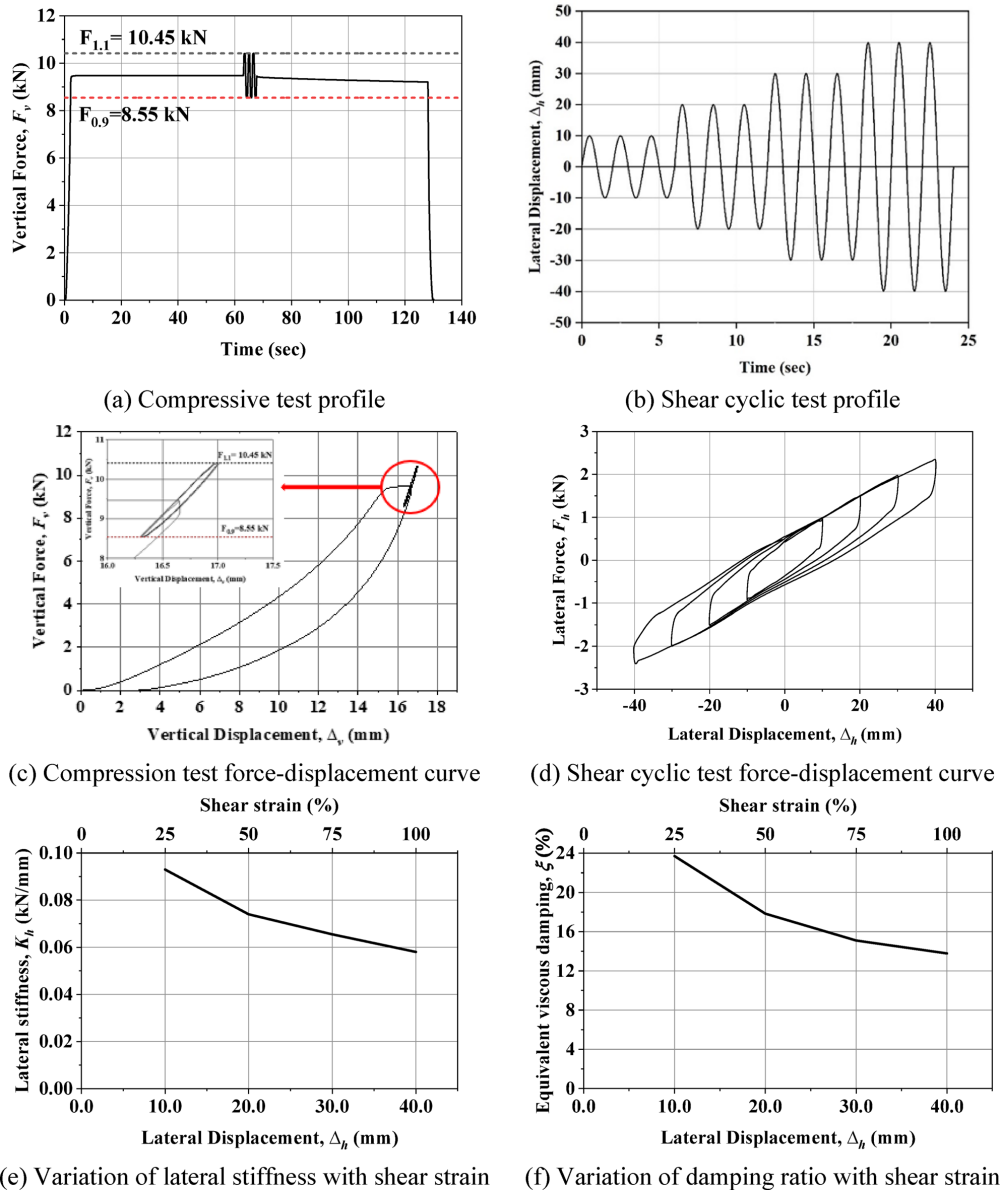
Input and output of the tests conducted on the UEI.

The compression test on the UEI was performed following the protocol established by Spizzuoco, where specimens were subjected to monotonic loading at a rate of 500 mm/min until reaching the specified design load^22^. This load was maintained for 60 s. Subsequently, three sinusoidal cycles with an amplitude of 10% of the design load were applied, as illustrated in Fig. 4(a). Afterward, a one-minute pause was introduced before unloading the specimens monotonically to zero load. The one-minute intervals between cyclic reversals were necessary to account for viscoelastic effects^22^. Figure 4(c) presents the load-displacement curves obtained from the test, which revealed significant nonlinearity in the UEI response.

Vertical stiffness is a key property that defines the relationship between the applied load and deformation along the isolator’s vertical axis. Maintaining consistent vertical stiffness is essential to ensure stable load transfer and prevent undesired dynamic responses in the isolated structure. Variations in vertical deformation among the isolators may lead to differential settlements, which could compromise structural integrity even in the absence of seismic activity. The isolator prototype underwent load cycles to assess the vertical stiffness ($\:{K}_{v}$), with the values for each cycle determined using Eq. 3.

In this Equation, when the design force with a 10% variation (1.1 times the design load and 0.9 times the design load) $\:{F}_{1.1}\:$and $\:{F}_{0.9}\:$are applied to the isolator, then the corresponding displacements $\:{\varDelta\:}_{1.1}\:$and $\:{\varDelta\:}_{0.9}\:$are recorded (refer to Fig. 4(c)), and this will be utilized to determine the vertical stiffness ($\:{K}_{v}$).

3 $$\:{K}_{v}=\frac{\left|{F}_{1.1}\right|+\left|{F}_{0.9}\right|}{\left|{\varDelta\:}_{1.1}\right|+\left|{\varDelta\:}_{0.9}\right|}.$$

Once the vertical stiffness $\:\left({K}_{v}\right)$ is determined, the vertical frequency ($\:{f}_{v}$) can be evaluated using Eq. 4, which incorporates vertical pressure ($\:P$), area and acceleration due to gravity $\:\left(g\right)$.

4 $$\:{f}_{v}=\frac{1}{2\pi\:}\sqrt{\frac{{K}_{v}\cdot\:g}{P\cdot\:A}}.$$

The vertical stiffness ($\:{K}_{v}$) of the UEI was determined by calculating the slope of the lines passing through the cyclic segments of the force-displacement curves. To obtain the dynamic vertical stiffness, the third sinusoidal curve was selected for analysis. The derived vertical stiffness values are documented in Table 4.

### Shear cyclic test

Shear cyclic experiments on elastomeric isolators provide a robust approach for precisely determining their stiffness and damping properties. These tests involve applying controlled horizontal displacements in conjunction with a vertical load. Notably, the experiments were conducted at standard room temperature, with data collected at a sampling rate of 200 Hz. The shear cyclic behavior was assessed by applying constant vertical pressure to the isolator while subjecting it to horizontal cyclic displacement. A sinusoidal waveform with three complete cycles was used to apply the displacement, with amplitudes incrementally varied to ± 25%, ± 50%, ± 75%, and ± 100% of the isolator’s thickness, as shown in Fig. 4(b). After each cycle, the isolator was vertically unloaded to ensure consistent testing conditions for each displacement amplitude. The horizontal force versus displacement curves are illustrated in Fig. 4(d).

Equation 5 is used to calculate the effective horizontal stiffness (*K*_*h*_) of the isolator for each amplitude cycle of the test.

5 $$\:{K}_{h}=\frac{\left|{F}_{h}^{+}\right|+\left|{F}_{h}^{-}\right|}{\left|{\varDelta\:}_{h}^{+}\right|+\left|{\varDelta\:}_{h}^{-}\right|}.$$

In this Equation, forces $\:{F}_{h}^{+}$and $\:{F}_{h}^{-}$ are obtained when the displacements $\:{\varDelta\:}_{h}^{+}$and $\:{\varDelta\:}_{h}^{-}$ are applied to the isolator. The aforementioned values are subsequently utilized to calculate *K*_*h*_. The equivalent viscous damping (*ξ*) of the UEI is determined using Eq. 6.

6 $$\:\xi\:=\:\frac{{W}_{d}}{2\pi\:{K}_{h}{{\varDelta\:}_{max}}^{2}}.$$

Here $\:{W}_{d}$ represents the area of the force-displacement curve obtained from the shear cyclic test, indicating the energy dissipated during the test. $\:{\varDelta\:}_{max}$ is the maximum input displacement given to the isolator during the shear cyclic test.

After calculating the horizontal stiffness $\:\left({K}_{h}\right),$ the isolation period ($\:{T}_{h}$), can be determined using Eq. 7, which incorporates vertical pressure ($\:P$), area $\:\left(A\right)$ and gravitational acceleration $\:\left(g\right)$.

7 $$\:{T}_{h}=2\pi\:\sqrt{\frac{P\cdot\:A}{{K}_{h}\cdot\:g}}.$$

When expressed in terms of shear modulus (*G*), Eq. 7 can be reformulated as follows:

8 $$\:{T}_{h}=2\pi\:\sqrt{\frac{P\cdot\:{t}_{r}}{G\cdot\:g}}.$$

The force-displacement graphs characterize two crucial mechanical properties: effective horizontal stiffness (*K*_*h*_) and equivalent viscous damping (*ξ*). The third cycle of each hysteresis loop from the test was used to evaluate effective horizontal stiffness across different strain levels. Similarly, the third cycle of each hysteresis loop was used to calculate the damping ratio. The variation of *K*_*h*_ and *ξ* with shear strain is shown in Fig. 4(e) & (f). Under a design load of 9.5 kN, the isolators exhibit a fundamental isolation period between 0.63 and 0.80 s as shear strain increases from 25 to 100%. It is important to note that as shear strain increases, the fundamental period of the isolated structure also increases. This shift, along with the associated changes in effective stiffness and energy dissipation, influences the overall isolation efficiency of the UEI. At 100% shear strain, the isolator’s isolation period is observed to be 0.8 s, whereas the initially specified design period was 0.95 s. This discrepancy arises due to variations in the shear modulus during the design process. Additionally, the design equations (Eq. 2) do not account for the nonlinear behavior of the UEI at higher shear strains. The effective shear modulus, calculated as 0.46 MPa under 100% shear strain (Eq. 2), is higher than the initial design shear modulus of 0.35 MPa. Therefore, selecting an appropriate shear modulus for the rubber is crucial in designing the UEI for buildings.

**Table 4:** Results of compression and shear Cyclic test on the UEI.

Description	Values
Vertical stiffness, $\:{K}_{v}$ (kN/mm)	2.73
Time Period (vertical), 1/$\:{f}_{v}$ (sec)	0.21
Natural frequency (vertical), $\:{f}_{v}$ (Hz)	4.6
Horizontal stiffness (100% strain), $\:{K}_{v}$ (kN/mm)	0.058
Time Period (horizontal) (100% strain), 1/$\:{f}_{v}$ (sec)	1.48
Natural frequency (horizontal) (100% strain), $\:{f}_{v}$ (Hz)	0.67
Equivalent viscous damping ratio (100% strain), *ξ* (%)	13.78

## Shake table test setup

The experiment was conducted using a shake table, sponsored by DST-SERB, capable of replicating capable of simulating actual earthquake acceleration time histories. A servo-hydraulic dynamic actuator system enabled the shake table to reproduce targeted earthquake acceleration histories. The acceleration responses of both the roof and the shake table were measured using accelerometers and laser displacement sensors, with data recorded via a compact data acquisition (DAQ) system. The uni-axial shake table, measuring 2 m × 3 m with a load capacity of 12 t, is equipped with a servo-hydraulic actuator capable of displacing the test model up to ± 75 mm linearly. The system can generate accelerations up to ± 3 g.

Uni-axial accelerometers (Model: 3055D3 by Kinemetrics Inc., USA), capable of detecting amplitudes ranging from ambient noise to ± 10 g, were used to capture acceleration responses. These accelerometers amplified and conditioned digital signals, transmitting the data directly to a compatible DAQ system. A 4-channel dynamic data acquisition system (m + p international, Germany) was used to collect accelerometer data, while two laser displacement sensors (Model: ILD1220, Micro-Epsilon, Germany) recorded model displacement during testing.

**Fig. 5:**
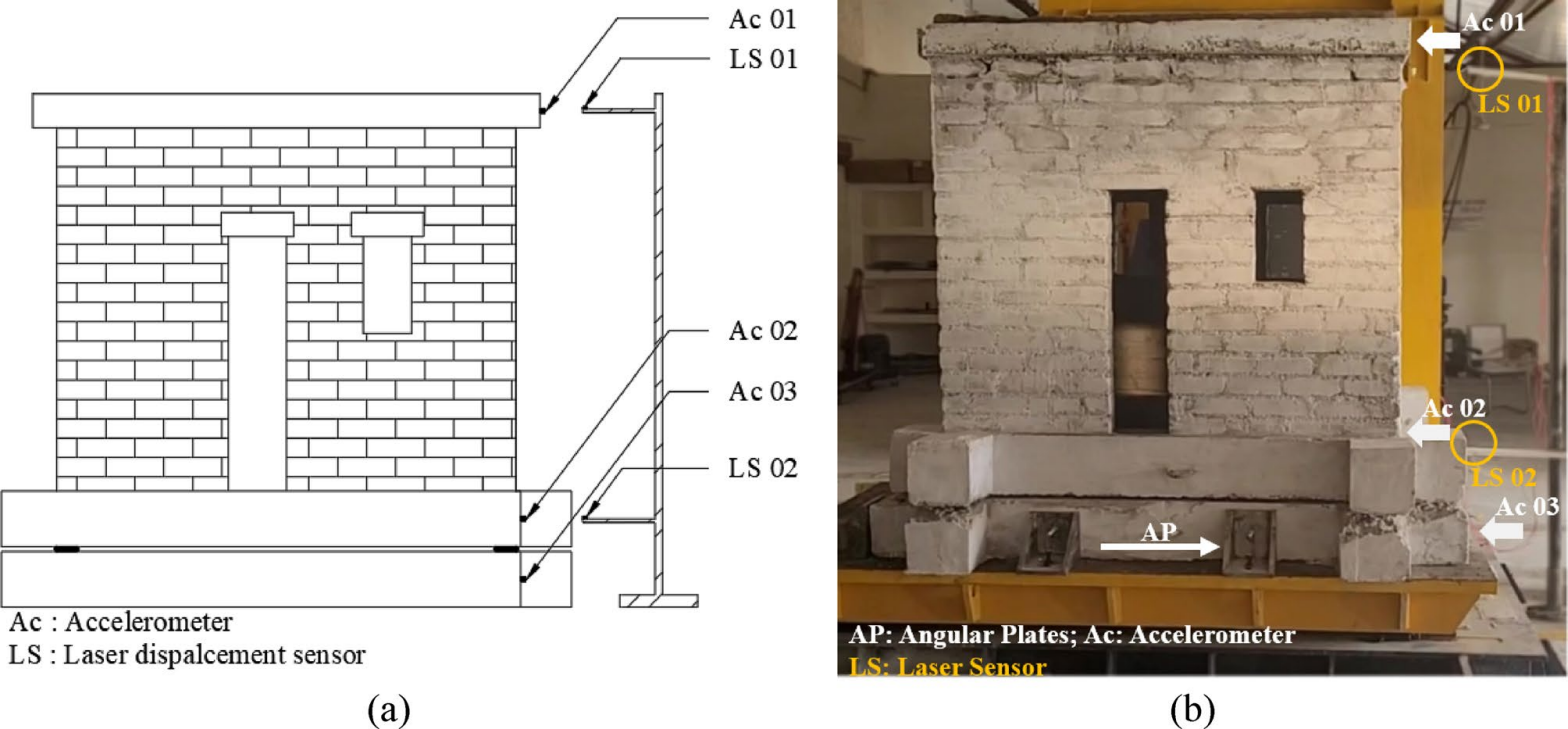
Location of accelerometers and laser displacement sensors connected to the masonry model. (a) Graphical; (b) Real.

The isolated half-scale masonry model is supported by four UEIs, one at each corner. The UEIs rest on the bottom plinth, which has dimensions similar to the top plinth used in the model’s construction. This design accounts for variations in friction coefficients between rubber-steel and rubber-concrete interfaces. The bottom plinth is secured to the shake table using slotted angular plates, as shown in Fig. 5(b). Additionally, Fig. 5(b) illustrates the configuration of the half-scale non-isolated model and its attachment to the shake table. Figure 2(a) shows the connection of the non-isolated model to the shake table.

**Fig. 6:**
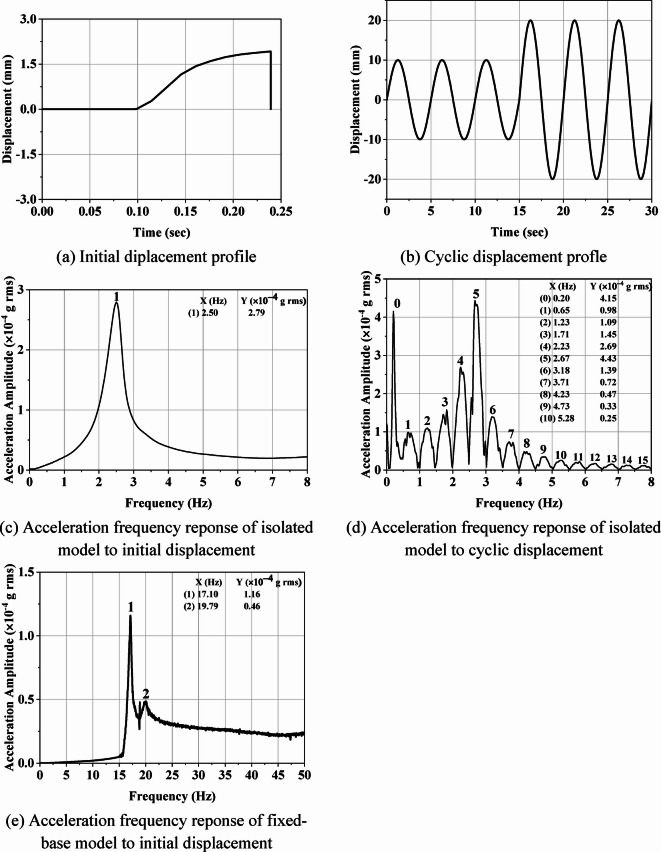
Vibration tests, shaketable input profile, isolated and fixed-base model output responses.

Uniaxial accelerometers are installed at various levels of the test model, as shown in Fig. 6. Accelerometer Ac 01 records acceleration at the shake table level, while accelerometers Ac 02 and Ac 03 measure acceleration at the plinth beam and roof levels, respectively. Laser displacement sensor placement, used to measure displacements at both the plinth and roof levels during earthquake simulations, is shown in Figs. 5(a) and (b).

### Free and forced vibration test

A free vibration test was conducted on the half-scale isolated masonry model using the shake table. An initial displacement of 2 mm was applied to the shake table at a frequency of 5 Hz, allowing the model to vibrate freely. The input profile is shown in Fig. 6(a). The fundamental natural frequency of the model was determined using an accelerometer, as shown in Fig. 6(c). The results indicate that the model’s time period was 0.4 s, whereas the isolation period for the system was 0.95 s. This discrepancy was attributed to the higher stiffness of the UEI at low shear strain levels. The design time period is intended to be achieved at 100% shear strain. At a 25% shear strain level (equivalent to a 10 mm displacement for the UEI), a time period of 0.51 s was observed (refer to Fig. 4(d)). Since the applied displacement during the free vibration test was 2 mm, less than the 10 mm required for the design time period, the measured 0.4 s time period aligns with expectations. To further assess the UEI’s performance relative to the design time period, a forced vibration test was conducted on the isolated half-scale masonry model. This test involved applying displacement amplitudes of 10 mm and 20 mm at a frequency of 0.2 Hz to the shake table, as shown in Fig. 6(b). Figure 6(d) illustrates how the UEI’s time period varies with increasing shear strain and cycle count. Notably, the initial peak (labeled as 0) in Fig. 6(d) corresponds to the forcing frequency applied, while the remaining peaks represent the natural frequencies of the isolated model from higher to lower strain levels, respectively. It can also be observed in the plot that the natural frequency of 1.23 Hz obtained at the third peak (labeled as 2 in Fig. 6(d)) approximately matches the natural frequency of 1.25 Hz obtained at 100% shear strain of UEI in the shear cyclic test (Fig. 4(d). Similarly, the remaining peaks (labeled as 3,4, etc.) correspond to the natural frequencies at varying shear strain levels during shaking. Since the test was conducted with increasing cycles and shear strain, distinguishing the frequencies associated with strain becomes challenging. It is important to note that increasing shear strain leads to an extended isolation period, affecting the isolation capacity of the UEI.

Additionally, a free vibration test was conducted on the non-isolated masonry building model using the same profile as for the isolated case (Fig. 6(a)). Figure 6(e) presents the frequency response obtained from this test. An accelerometer was placed on the slab along the *x*-axis to measure frequency response in the *z*-direction. The first fundamental frequency recorded was 17.10 Hz along the *x*-axis, corresponding to a time period of 0.058 s, and the second frequency was 19.79 Hz along the *z*-axis, corresponding to a time period of 0.05 s. Additionally, an impact hammer test was conducted on the non-isolated building model to cross-verify the obtained frequencies. The natural frequencies obtained from the impact hammer test are similar to those obtained by applying initial displacement.

Following the free and forced vibration tests, additional shake table tests were conducted using seven earthquake ground motions sourced from the PEER strong-motion database, with the key characteristics outlined in Table 4. The subsequent sections discuss the shake table tests performed on the masonry models.

### Ground motions for the shake table test

The isolation period for the building was determined as a balance between achieving a longer period to enhance spectral acceleration mitigation and ensuring the stability of the isolation system. This consideration resulted in an isolation period of 0.95 s, positioning the system within the displacement-sensitive region where spectral acceleration is approximately equal to the peak ground acceleration (PGA). The corresponding design displacement of the isolated system, based on the maximum considered earthquake (MCE) scenario, is 40 mm, with an equivalent damping ratio of 15%.

For the shake table tests, seven ground motions were selected from the PEER Strong Ground Motion Database^42^. The selection criteria included earthquake events with a moment magnitude (M_w_) between 5.0 and 6.5 and an epicentral distance within the range of 7.5 km to 15 km, allowing for a 10% tolerance above and below the average spectral response in the period range of 0.1 to 1.0 s.

Table 5 provides a summary of the selected ground motions, while Fig. 1-A (Appendix) presents the acceleration time history. To ensure compatibility with actuator constraints, the chosen ground motions were adjusted to maintain a peak ground displacement within approximately ± 43 mm.

**Table 5:** Description of earthquake records.

S. No.	Earthquake	Station	Date	PGA (g)	PGD (mm)
1	Friuli	TOLMEZZO	06/05/1976	0.35	40.67
2	Oroville	RSN 112	8/8/1975	0.31	40.40
3	Bigbear	RSN 901	6/28/1992	0.48	43.40
4	Palm Spring	RSN 517	7/8/1986	0.27	30.98
5	Gilroy	RSN 2020	5/14/2002	0.43	21.80
6	Parkfield	RSN 4134	9/28/2004	0.31	37.01
7	Ramage Ranch	RSN 19,043	6/20/2009	0.37	17.77

PGA: Ipeak ground acceleration; PGD: Ipeak ground displacement.

### Shake-table testing

The shake table test of a scaled URM building model subjected to a range of earthquake excitations is discussed. Responses were recorded at multiple building levels at a 128 Hz sampling rate. The URM structure exhibits low resistance when subjected to ground motion, making it initially unclear how much acceleration it can sustain before incurring any damage. To address this, the isolated masonry model was tested first by keeping it on four UEIs supported on the bottom plinth (Fig. 5). The test was then repeated for the non-isolated masonry model by directly fixing the top plinth on the shake table to simulate a fixed condition (Fig. 2).

The experimental results from shake table excitations along the *x*-axis of the model using the seven earthquake motions are discussed. Table 6 displays the peak acceleration and peak displacement at various levels of the model in response to the earthquake excitations. It has been observed that the peak acceleration recorded from the shake table exceeds the input acceleration. The input acceleration time history is shown in Fig. 1-A (Appendix). The comparison of the spectrum curve for each ground motion is also compared in Fig. 2-A (Appendix). This discrepancy arises from the inherent limitations of the shake table’s various capacities, including maximum displacement, velocity, frequency, and oil supply constraints^43,44^. To mitigate this issue, the shake table should undergo calibration using an iterative approach, namely adaptive inverse control^45^. This calibration, commonly referred to as tuning, optimizes signal reproduction by adjusting control parameters. Given the dynamic interaction between the test model and the shake table, this tuning process should be performed with the model on the shake table. However, this procedure carries a significant risk of inducing low-level fatigue damage to the model^46^, altering its behavior even before testing. As a result, the test was conducted without implementing the tuning process. In the rest of the paper, the results were compared with those of shake table-induced acceleration.

The data in Table 6 reveals a significant reduction in peak acceleration at the base beam level across all excitation cases. Additionally, the peak acceleration values at the plinth and roof levels are nearly identical, suggesting that the isolated model did not experience notable acceleration amplification along its height. This indicates that the structure above the isolation level behaves as a rigid body, and no significant seismic forces were generated, allowing the URM model to endure the full intensity of seismic excitations without damage.

**Table 6:** Peak acceleration and relative displacement at different levels of the isolated model.

	Peak acceleration (g)			Peak relative displacement (mm)		
Earthquake	Shake table	Plinth level	Roof level	Top Plinth level	Roof level	Rocking displacement
Friuli	0.80	0.28	0.31	12.82	14.91	1.2
Oroville	0.76	0.32	0.36	17.37	19.95	1.07
Bigbear	0.60	0.28	0.30	13.09	15.20	1.67
Palm Spring	0.56	0.26	0.27	11.62	12.95	2.18
Gilroy	0.39	0.19	0.20	8.81	9.31	1.52
Parkfield	0.34	0.20	0.23	7.53	9.31	1.88
Ramage Ranch	0.35	0.30	0.31	14.61	14.26	1.98

**Fig. 7:**
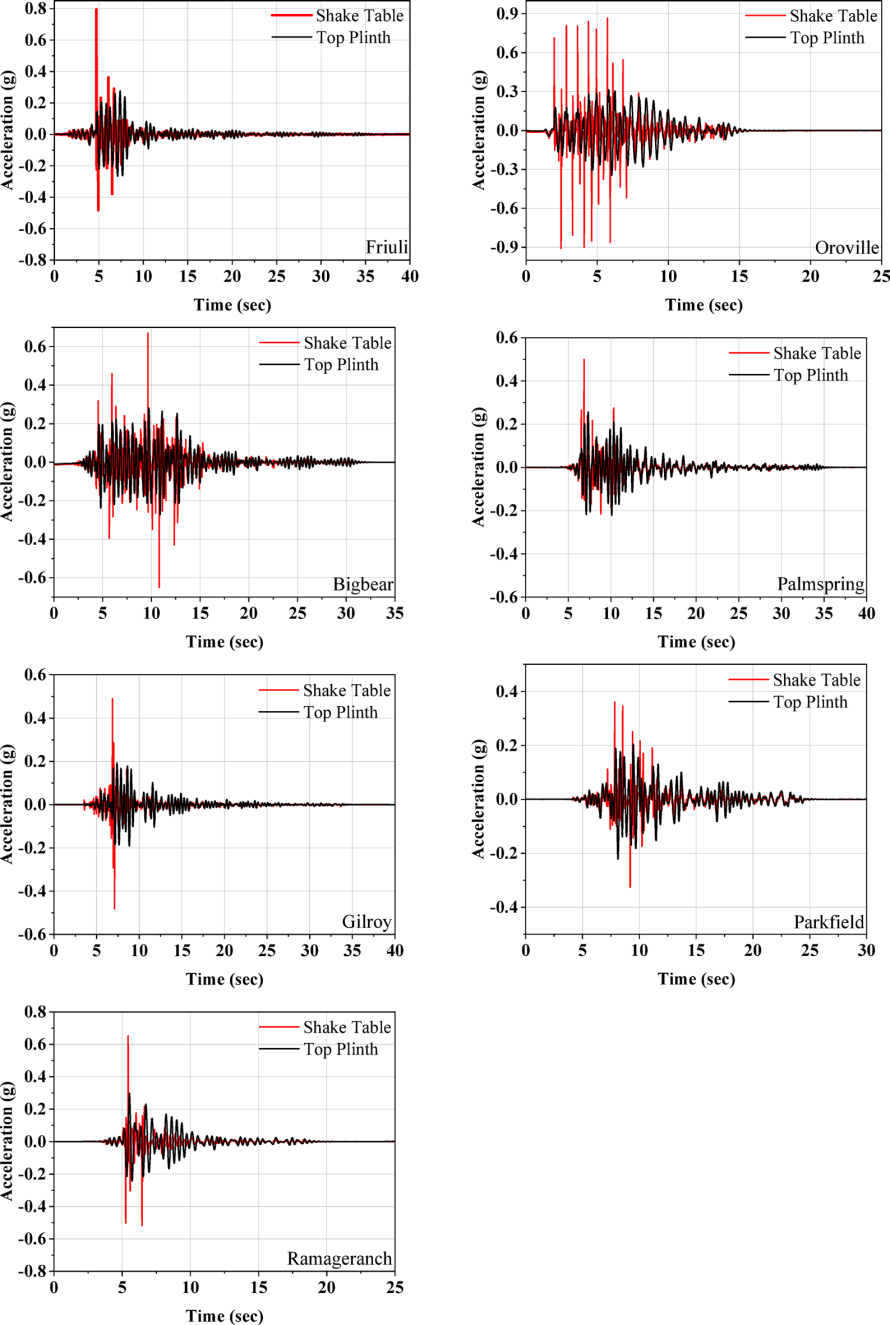
Acceleration response at shake table and plinth beam of the isolated model.

Figure 7 illustrates the acceleration responses recorded at the shake table and the top plinth level. The data reveals that the peak acceleration at the base beam level is reduced by 65%, 64%, 69%, 40%, 48%, 47%, and 57% for the Friuli, Oroville, Bigbear, Palm Spring, Gilroy, Parkfield, and Ramage Ranch earthquakes, respectively. The effectiveness of the UEI in seismic isolation improves with higher acceleration levels.

**Fig. 8:**
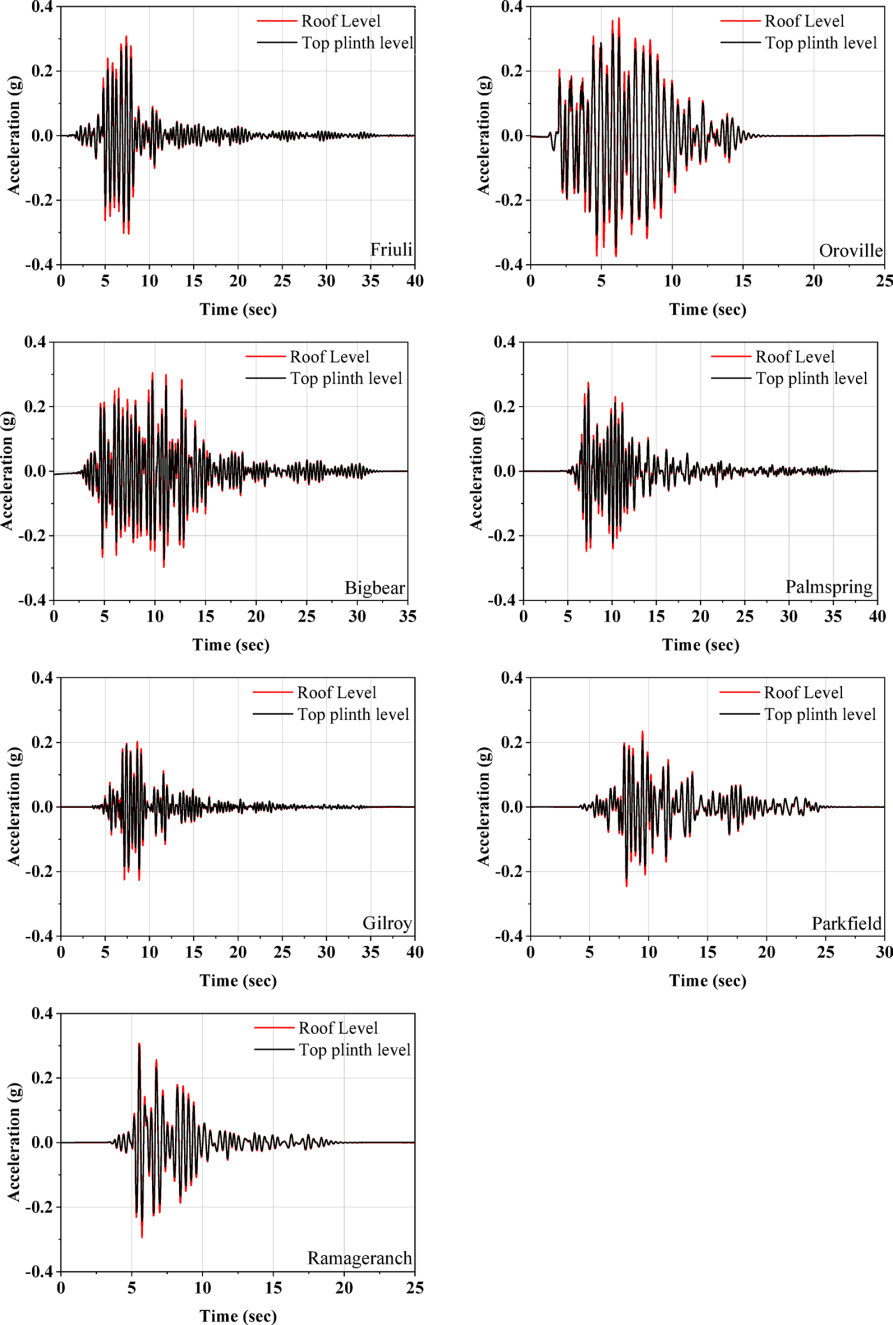
Acceleration response at the top plinth level and roof level of the isolated model.

**Fig. 9:**
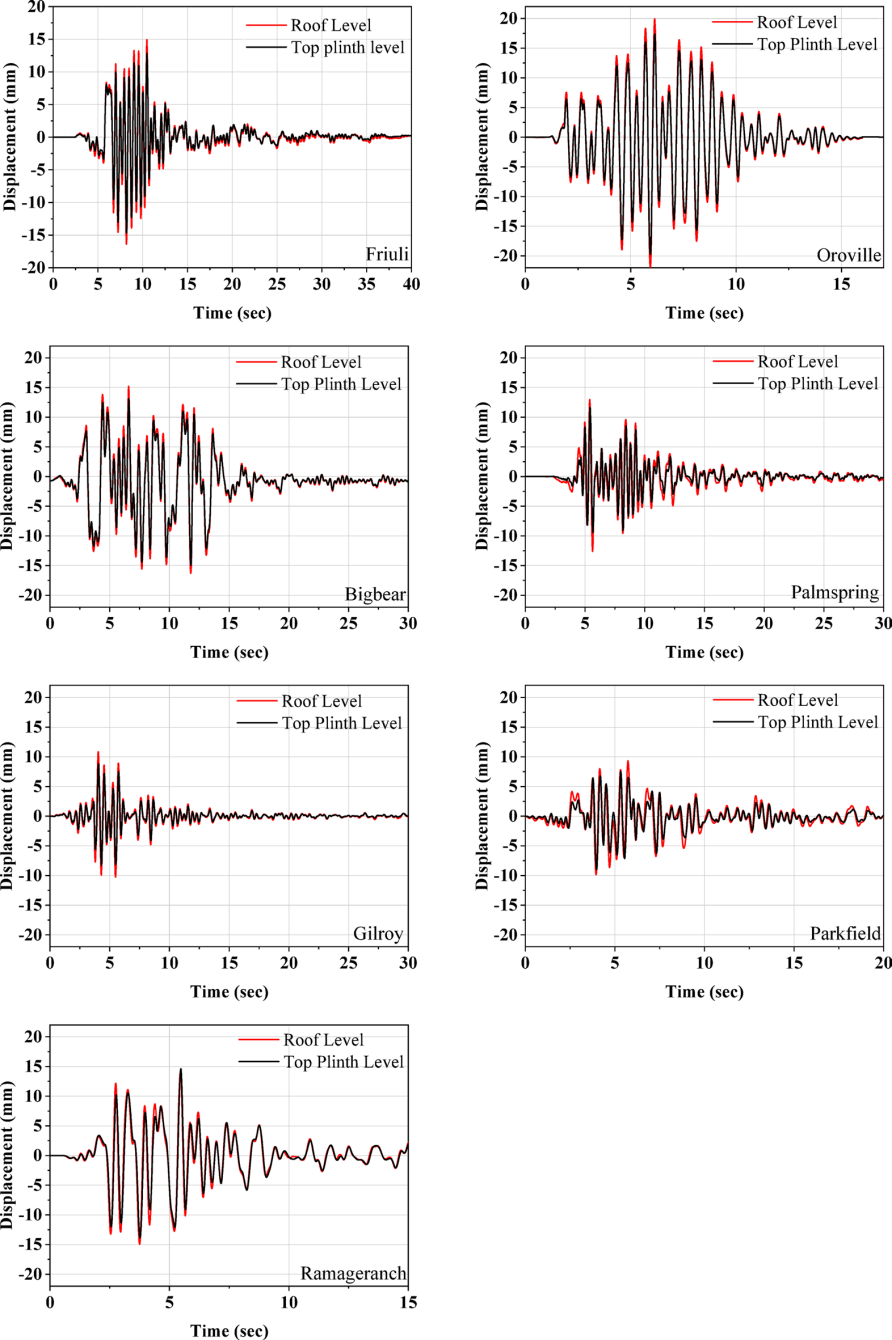
Displacement response at the top plinth level and roof level of the isolated model.

Further, Fig. 8 plots the acceleration responses recorded at both the top plinth and roof levels. The acceleration time history demonstrates that there is no significant amplification along the height of the half-scale masonry model for any of the earthquakes. This indicates that the UEI is providing sufficient isolation to the masonry model, leading to the almost rigid body motion of the superstructure. The displacement responses at the top plinth and roof levels are compared in Fig. 9. The maximum lateral displacement observed at the isolator is 19.95 mm during excitation by the Oroville earthquake. For this event, the roof level displacement recorded is 17.37 mm, with a relative displacement of 2.58 mm, resulting in a drift of 0.17% for a model height of 1500 mm. The test model consistently exhibited minimal acceleration amplification along its height and low story drift.

**Fig. 10:**
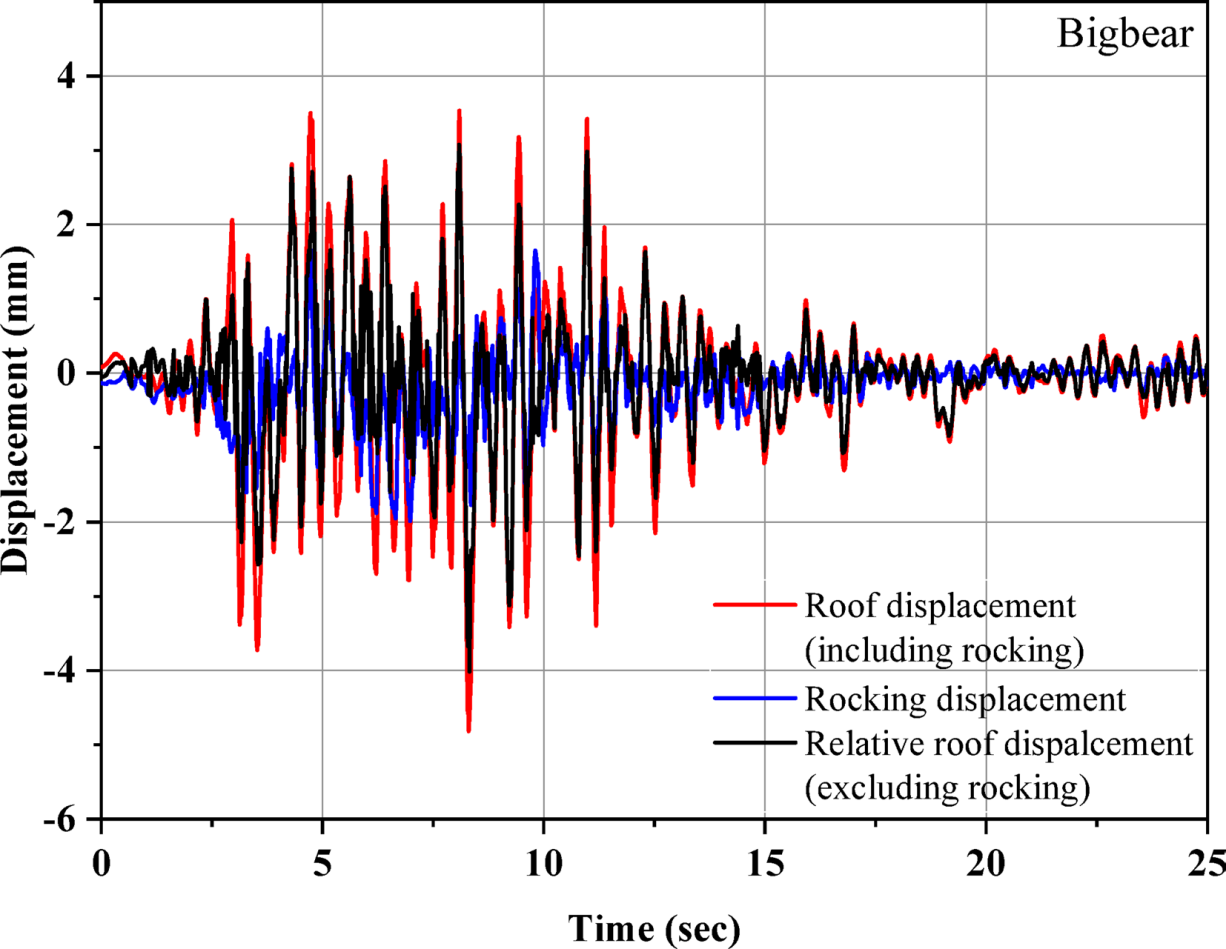
Roof displacement with and without the rocking displacement of the isolated model.

This relative displacement includes the rocking displacement observed during the test. To measure rocking displacements, vertical displacements at the ends of the half-scale model were recorded using laser displacement sensors. Figure 10 compares the roof displacement with the rocking displacement during the Bigbear earthquake. A maximum rocking displacement of 1.98 mm was observed during the ground motion. Despite the higher vertical flexibility of the UEI compared to reinforced bearings, the rocking displacement accounts for less than 42% of the peak roof displacement. This finding supports previous studies indicating that a vertical frequency greater than 3 Hz minimizes rocking motion, leading to a primary horizontal deflection mode of the structure. The maximum rocking displacements for the various earthquakes are summarized in Table 6. The rocking displacement doesn’t contribute to any lateral deformation associated with the structural stiffness. Additionally, it was noted that after each test, the UEI and the isolated model returned to their original positions, with the UEI remaining stable throughout the test, and no instability was observed in the model. This shows that UEI has not undergone any permanent deformation. In addition, the friction between the concrete and the top and bottom rubber surface of UEI does not allow sliding, helping the building to remain in its original position even after a seismic event.

## Isolated and non-isolated models’ comparison

**Fig. 11:**
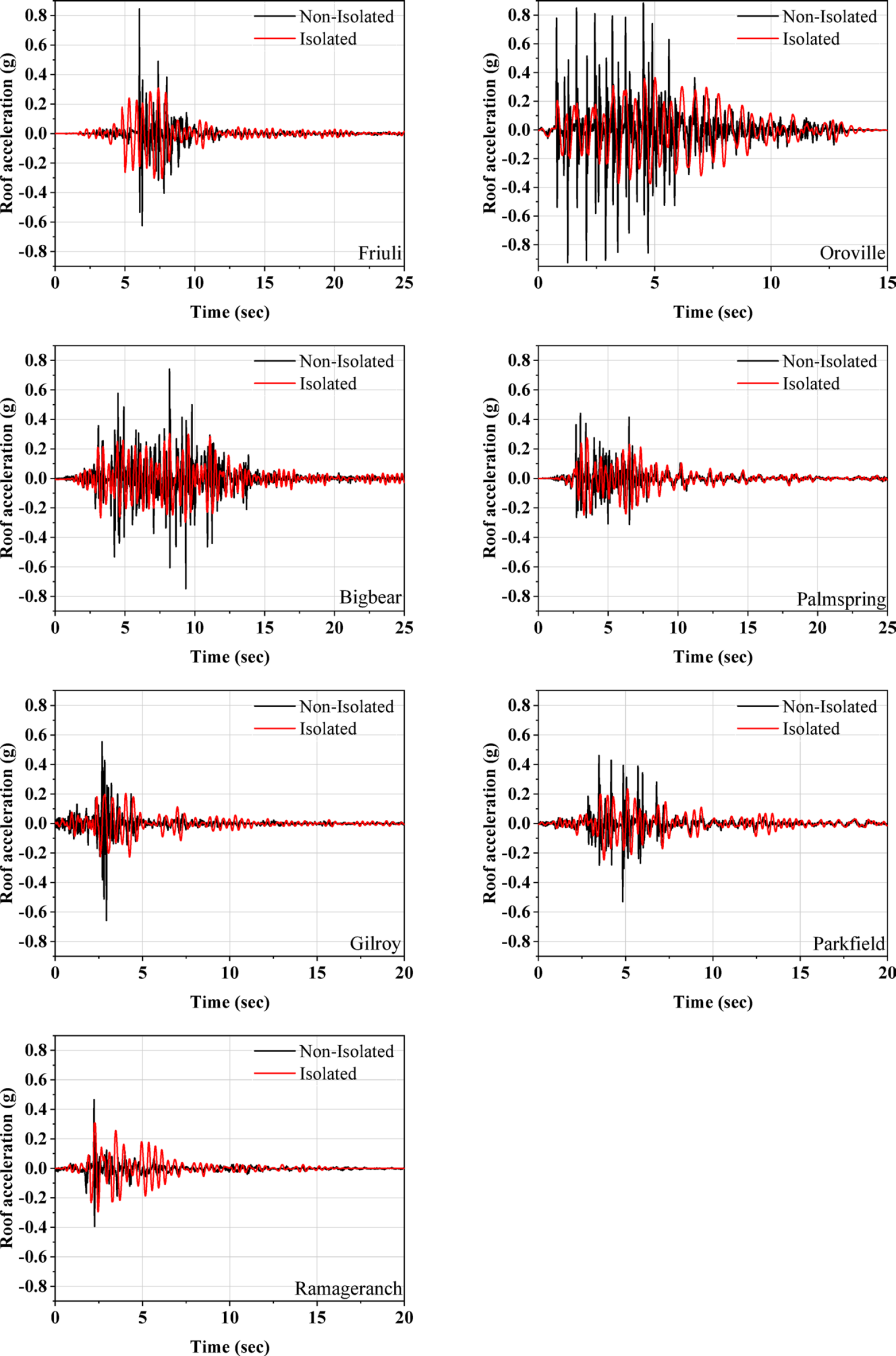
Comparison of roof acceleration for isolated and non-isolated models.

This section presents a comparative analysis of various dynamic parameters for both isolated and non-isolated models under the influence of seven distinct seismic events. Figure 11 illustrates the acceleration time histories for both model types. Notably, during the Friuli earthquake, the roof-level acceleration of the non-isolated model was found to be 2.7 times greater than that of the isolated model. The minimum acceleration amplification at the roof level for the non-isolated model was recorded during the Ramage Ranch earthquake, which was 1.5 times the acceleration observed in the isolated model. Generally, accelerations were amplified in the non-isolated model, with the rate of increase being more pronounced along the height of the structure. In contrast, the isolated model did not exhibit such amplification of accelerations along its height. Furthermore, it was observed that as the earthquake intensity increased, the reduction in roof acceleration for the isolated model became more significant. Consequently, the effectiveness of the isolation system appears to be enhanced by higher seismic intensities.

**Fig. 12:**
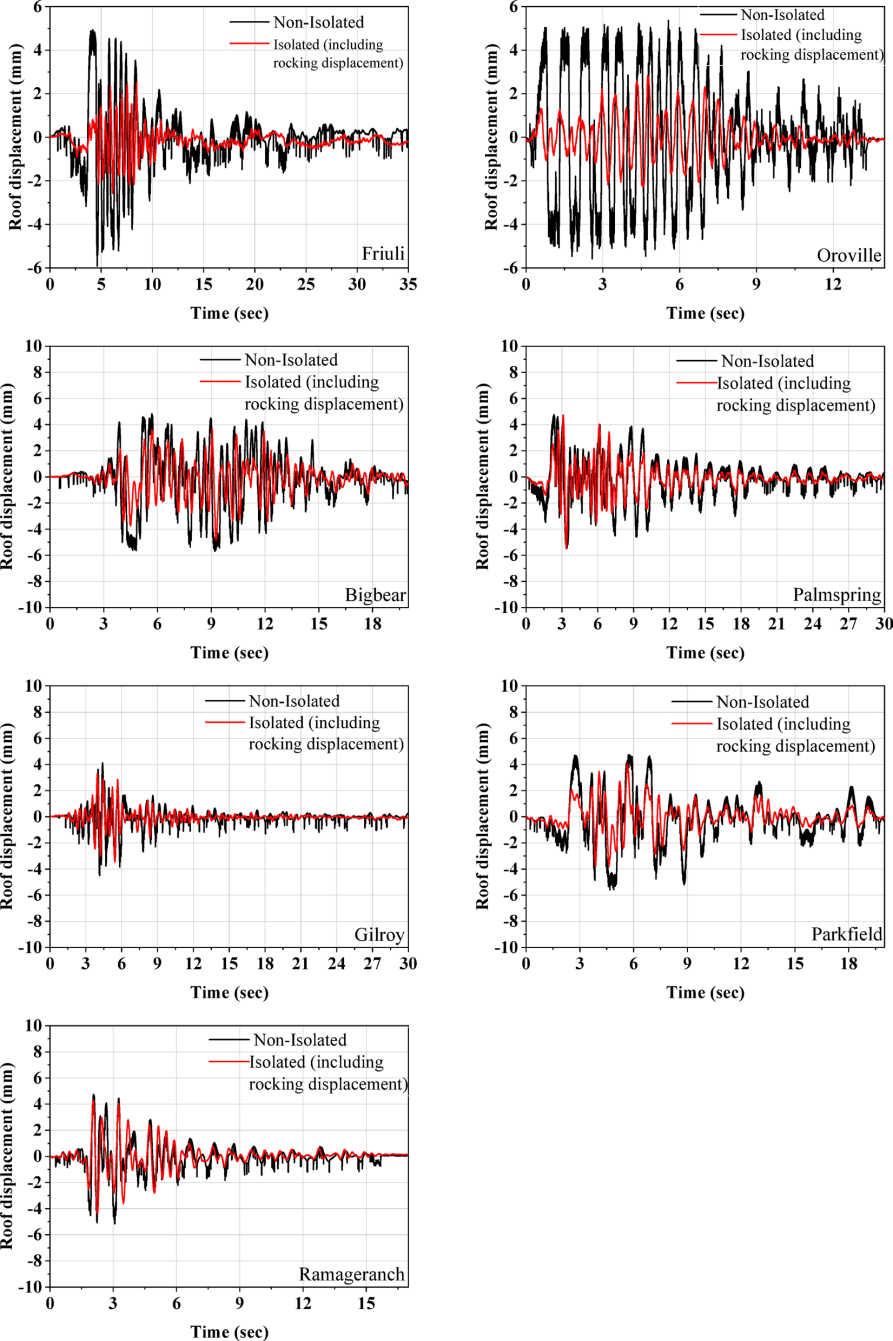
Comparison of roof displacement for isolated and non-isolated models.

**Fig. 13:**
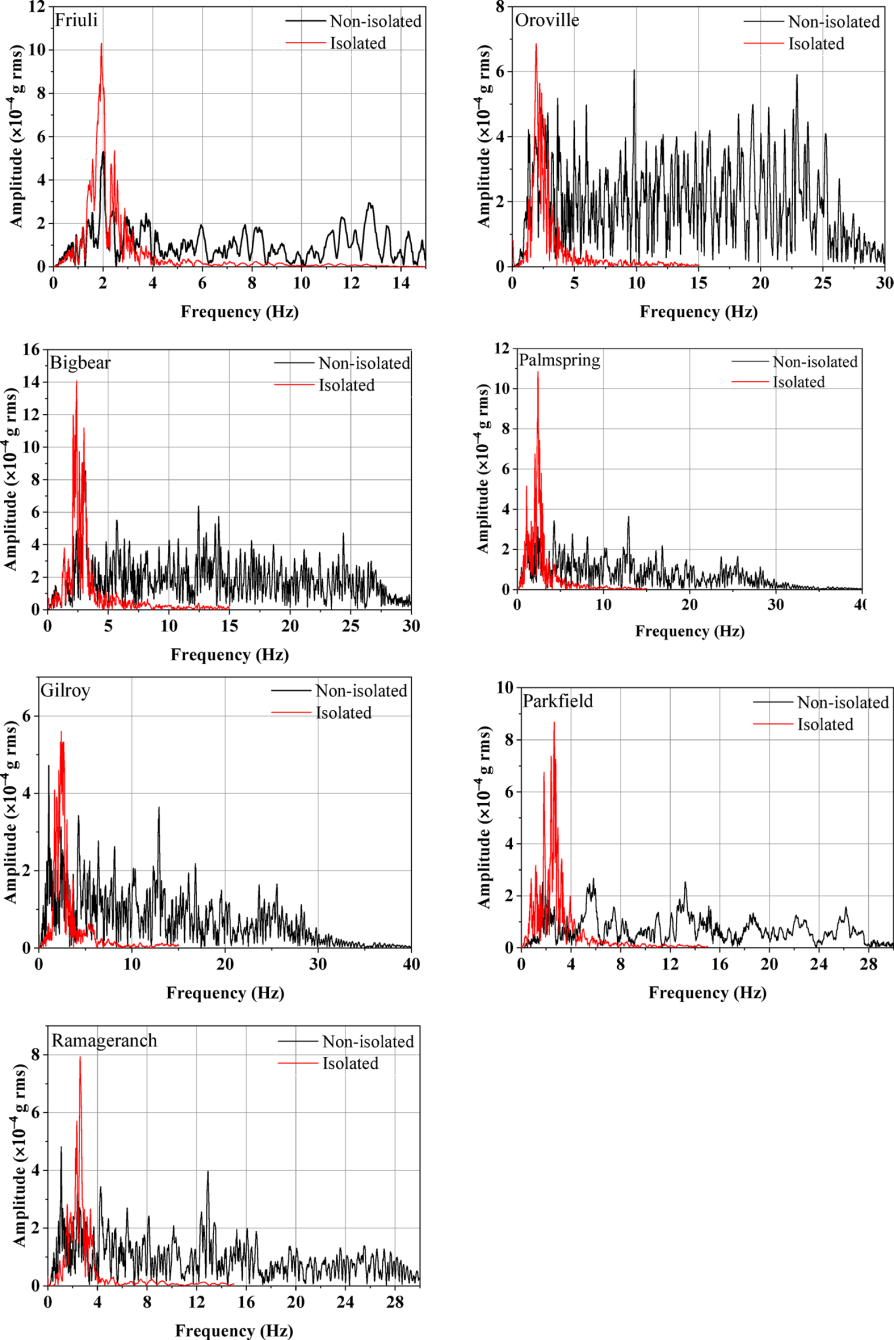
Comparison of acceleration frequency responses for isolated and fixed-base models.

Figure 12 illustrates the displacement time histories for both isolated and non-isolated models. For the isolated model, the reported relative roof displacement includes a contribution from rocking motion. Consequently, the actual displacement in the isolated half-scale masonry model is lower than the recorded relative displacement. Despite this, the relative roof displacement of the isolated model is still consistently lower than that of the non-isolated model across all ground motions. The observed reductions were 49%, 64%, 25%, 1%, 20%, 15%, and 10% for the Friuli, Oroville, Bigbear, Palm Spring, Gilroy, Parkfield, and Ramage Ranch earthquakes, respectively. The observed trend is that greater reductions in relative roof displacement are achieved under higher-intensity ground motions. At higher input intensities, the increased seismic demand leads to larger shear strains in the isolators. Under such conditions, the isolators exhibit lower stiffness (Refer to Fig. 4(e). In contrast, for lower-intensity ground motions, the isolators operate mostly in their initial (stiffer) range, where the displacement and strain levels are not sufficient to engage their full isolation potential. Table 7 compares the maximum base shear and maximum overturning moments for both isolated and non-isolated half-scale masonry models. The peak base shear was calculated by multiplying the peak acceleration recorded at the base of the masonry model by its total weight. For the overturning moment, shear forces at different heights along the masonry model were determined based on acceleration data recorded at those respective levels. These shear forces were then multiplied by their corresponding vertical distances (from the base) to compute the overturning moment contributions, and the peak value was reported. The non-isolated model consistently exhibits much higher peak base shear than the isolated model across all earthquakes. Additionally, the overturning moments are significantly greater in the non-isolated model for all seven input earthquakes. These findings demonstrate the effectiveness of the base isolation system developed in this study, with performance improving further at higher levels of induced displacement at the UEIs.

The fundamental frequency of the isolated half-scale model during the earthquake is derived from the recorded roof acceleration. The M + P International SO analyzer software facilitates the conversion of time response data to frequency response. To compare the frequency response of the isolated and non-isolated masonry models, the roof acceleration time histories were converted to the frequency domain using Fourier analysis. As shown in Fig. 13, the resulting spectra of the non-isolated model exhibit dominant peaks across a broader frequency range, typically from 0 to 30 Hz, depending on the input ground motion. In contrast, the isolated model shows a clear concentration of spectral energy within the lower frequency band of 0 to 3 Hz. This shift in dominant frequency is attributed to the increased flexibility introduced by the UEI, which reduces the overall stiffness of the system.

**Table 7:** Comparison of maximum base shear and the maximum overturning moment.

Earthquake	Maximum Base Shear (kN)		Maximum overturning moment (kN-m)	
	Isolated	Non-isolated	Isolated	Non-isolated
Friuli	11.39	31.59	11.24	30.74
Oroville	13.35	32.56	13.32	32.89
Bigbear	11.18	26.20	10.94	26.68
Palm Spring	10.20	19.02	9.90	17.04
Gilroy	8.14	21.86	8.22	23.02
Parkfield	8.73	19.85	8.89	19.36
Ramage Ranch	11.74	15.96	11.37	16.55

This demonstrates the effectiveness of the UEI for the half-scale masonry model in reducing seismic demands. Further investigations are required to evaluate the behavior of UEIs under varying earthquake intensities, different target isolation periods, and their influence on rocking motion. In addition, long-term performance aspects, such as material degradation and its effect on the frictional interface between the plinth and UEI, cyclic fatigue, and environmental durability, remain to be explored through extended cyclic and aging tests.

## Conclusion

A single-story, half-scale, unreinforced fly ash brick model with the UEI was subjected to seven different ground motions on the shake table. The primary objective of this study is to demonstrate that the use of low-cost UEIs can reduce the vulnerability of URM structures. The observed reduction in amplification of roof acceleration along its height supports this objective. Additionally, the model did not undergo any damage even under high-intensity earthquake conditions, further validating the effectiveness of the UEI in enhancing seismic resilience.

The same model was also tested by placing it directly on the shake table and subjecting it to ground motion. The responses obtained from this setup were compared with those of the isolated model. The isolated model exhibited reductions in roof acceleration, roof displacement, maximum base shear, and maximum overturning moment compared to the non-isolated model.

The conclusions drawn from this shake table study are summarized below:

- The proposed Unreinforced Elastomeric Isolators (UEIs) demonstrated high effectiveness in reducing seismic demands in a half-scale unreinforced masonry (URM) model. Notably, roof acceleration was reduced by up to 65% across a range of ground motions.
- The isolation system’s efficiency was found to be strain-dependent. As lateral displacement increased, the effective stiffness decreased, resulting in a longer vibration period and improved isolation performance. For instance, the isolation period of 0.8 s was achieved with the shear strain of 100%.
- The rocking contribution to the total roof displacement in the isolated model was observed to be minimal. Even with rocking included, the relative roof displacement in the isolated case was up to 49% lower than in the non-isolated model for higher intensity ground motions.
- Higher frequencies associated with the non-isolated masonry model are attenuated to a lower frequency range using UEI.
- A maximum reduction of 64% in base shear and in overturning moment was recorded in the isolated model compared to the fixed-base model, confirming the isolator’s effectiveness in reducing seismic force transmission.
- There is significant potential for incorporating UEIs in URM buildings to mitigate seismic vulnerability. Installing UEIs at the interface between the superstructure and substructure of URM buildings is straightforward, cost-effective, and hassle-free. This approach facilitates the adoption of this technology in less developed countries experiencing high seismic activity with additional investigations.

## Electronic supplementary material

Below is the link to the electronic supplementary material.

Supplementary Material 1

## Data Availability

Some or all data, models, or code that support the findings of this study are available from the corresponding author upon reasonable request.
