# Supplementary material for: Shake-table testing of unreinforced fly ash brick masonry model with unreinforced elastomeric isolator
Source: Sci Rep. 2025 Jun 4;15:19650. doi: 10.1038/s41598-025-04549-5 (PMC12137839; doi:10.1038/s41598-025-04549-5)
Supplement: Supplementary file 1 — Supplementary Material 1 [file 41598_2025_4549_MOESM1_ESM.docx]

Appendix: Input acceleration and the comparison of Acceleration spectra.

|  |  |
| --- | --- |
|  |  |
|  |  |
|  |  |
| **Fig. 1-A** Illustration of ground acceleration input earthquake records | |

|  |  |
| --- | --- |
|  | 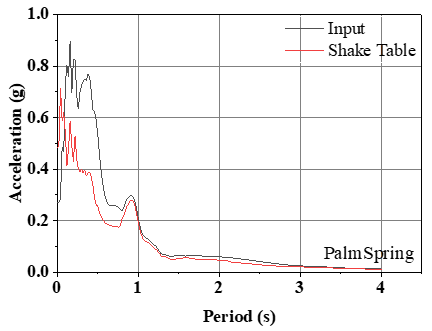 |
|  |  |
|  |  |
| **Fig. 2-A** Comparison of Response spectra of Input and shake table acceleration | |
